# Pre- and post-synaptic roles for DCC in memory consolidation in the adult mouse hippocampus

**DOI:** 10.1186/s13041-020-00597-2

**Published:** 2020-04-07

**Authors:** Stephen D. Glasgow, Edwin W. Wong, Greta Thompson-Steckel, Nathalie Marcal, Philippe Séguéla, Edward S. Ruthazer, Timothy E. Kennedy

**Affiliations:** 1 Montréal Neurological Institute, Department of Neurology & Neurosurgery, McGill University, 3801 Rue University, Montréal, Québec H3A 2B4 Canada; 2 NSERC CREATE Neuroengineering Training Program, McGill University, Montréal, Canada; 3 Department of Anatomy and Cell Biology, McGill University, 3640 Rue University, Montreal, Quebec H3A 0C7 Canada

**Keywords:** CA1 pyramidal neurons, Schaffer collaterals, Long-term potentiation, Axon guidance, Spatial memory, SHANK, PSD-95, S6, p34-arc, Arp2/3, Guidance cues, Netrin-1, Learning, Deleted-in-colorectal cancer

## Abstract

The receptor deleted in colorectal cancer (DCC) and its ligand netrin-1 are essential for axon guidance during development and are expressed by neurons in the mature brain. Netrin-1 recruits GluA1-containing α-amino-3-hydroxy-5-methyl-4-isoxazolepropionic acid receptors (AMPARs) and is critical for long-term potentiation (LTP) at CA3-CA1 hippocampal Schaffer collateral synapses, while conditional DCC deletion from glutamatergic neurons impairs hippocampal-dependent spatial memory and severely disrupts LTP induction. DCC co-fractionates with the detergent-resistant component of postsynaptic density, yet is enriched in axonal growth cones that differentiate into presynaptic terminals during development. Specific presynaptic and postsynaptic contributions of DCC to the function of mature neural circuits have yet to be identified. Employing hippocampal subregion-specific conditional deletion of DCC, we show that DCC loss from CA1 hippocampal pyramidal neurons resulted in deficits in spatial memory, increased resting membrane potential, abnormal dendritic spine morphology, weaker spontaneous excitatory postsynaptic activity, and reduced levels of postsynaptic adaptor and signaling proteins; however, the capacity to induce LTP remained intact. In contrast, deletion of DCC from CA3 neurons did not induce detectable changes in the intrinsic electrophysiological properties of CA1 pyramidal neurons, but impaired performance on the novel object place recognition task as well as compromised excitatory synaptic transmission and LTP at Schaffer collateral synapses. Together, these findings reveal specific pre- and post-synaptic contributions of DCC to hippocampal synaptic plasticity underlying spatial memory.

## Introduction

Long-term potentiation (LTP) is an extensively studied form of activity-dependent synaptic plasticity [1]. Brief high-frequency stimulation (HFS) of Schaffer collateral synapses, the primary excitatory efferent connection between CA3 and CA1 pyramidal neurons in the hippocampus, results in a long-lasting change in the strength of synaptic transmission that is mediated primarily through the modification and membrane recruitment of excitatory postsynaptic receptors on dendritic spines [2, 3]. Accordingly, alteration of dendritic spine structure and synaptic composition can impact neuronal transmission and activity-dependent plasticity.

First described in the embryonic central nervous system, the secreted chemotropic guidance cue, netrin-1, regulates cytoskeletal reorganization, mediates cell adhesion, and directs cell and axon migration [4–6]. In the mature nervous system, conditional deletion of netrin-1 from principal excitatory forebrain neurons impairs activity-dependent plasticity, and bath application of exogenous netrin-1 results in rapid synaptic recruitment of Ca^2+^-permeable AMPARs [7]. Further, hippocampal-dependent spatial memory is impaired in mice that conditionally lack netrin-1 expression by principal excitatory forebrain neurons [8], suggesting that netrin-1 signaling has a critical role in synaptic plasticity underlying hippocampal-dependent spatial memory.

The canonical netrin-1 receptor, deleted in colorectal cancer (DCC), is essential for neural development and widely expressed in the mature nervous system [4, 9]. DCC null mice die within a few hours following birth, making it impossible to study DCC loss-of-function in adults using conventional knockouts [10]. To investigate the specific functional role of DCC at presynaptic and postsynaptic sides of the Schaffer collateral synapse in the mature brain, we removed a floxed *dcc* allele using subregion-specific Cre recombinase driver mouse lines. Selective deletion of DCC from CA1 glutamatergic neurons increased thin-type dendritic spines with concomitant reduction in mushroom-type dendritic spines, reduced levels of postsynaptic adaptor and signaling proteins, decreased amplitude of spontaneous excitatory postsynaptic currents, and impaired spatial memory, but resulted in no change in HFS-induced LTP. In contrast, mice selectively lacking DCC expression by CA3 glutamatergic neurons showed impaired spatial memory accompanied by reduced basal excitatory synaptic transmission and attenuated HFS-induced LTP. Our findings suggest that these deficits may be due to reduced DCC-mediated presynaptic vesicular mobilization in CA3 neurons, and reveal distinct pre- and postsynaptic functions for DCC at Schaffer collateral synapses contributing to synaptic plasticity underlying memory consolidation in the adult brain.

## Materials and methods

### Animals

R4ag11(CaMKIIα)-Cre [11] mice were generously provided by Dr. Scott Zeitlin (University of Virginia). Grik4-Cre [12] mice were obtained from Jackson Laboratory (Bar Harbor, ME, USA) and maintained on a C57BL/6 J genetic background. Both lines were crossed with mice homozygous for a floxed *dcc* allele, DCC^*fl/fl*^ [13, 14]. *Cre* recombinase is detectable at P17 and P14 in R4ag11-Cre and Grik4-Cre, respectively [11, 15]. Significant reduction of DCC protein levels in R4ag11 CaMKIIα-Cre- DCC^*fl/fl*^ (CA1 DCC cKO) and Grik4-Cre- DCC^*fl/fl*^ (CA3 DCC cKO) mice was observed by 6 months of age, therefore all experiments were performed with mice at least 6 months old. Both males and females were used in behavioural, electrophysiological, and immunohistochemical experiments. We observed no statistically significant differences between sexes, and therefore all data were pooled for analysis. Control experiments were performed using littermates that were negative for *Cre* and homozygous for floxed alleles of *dcc*.

### Immunohistochemistry

Immunohistochemistry was performed on sections of brain tissue including the hippocampus from either R4ag11-Cre/DCC^*fl/fl*^ or Grik4-Cre/DCC^*fl/fl*^ mice. Briefly, mice were deeply anaesthetized by intraperiotoneal injection of a mixture of 2,2,2 – tribromoethyl alcohol and tert-amyl alcohol diluted at 2.5% in PBS, and transcardially perfused with cold PBS (pH: 7.4) followed by 4% paraformaldehyde (PFA). Brains were isolated and immersed in 4% PFA diluted in cold PBS for 24 h at 4 °C, followed by 24 h cryoprotection in 30% sucrose in PBS. Brains were frozen and sections cut using a cryostat (Leica CM1850). Antigen retrieval was performed by boiling sections in 0.1 M citrate buffer for 10 mins, allowed to cool to RT and subsequently washed three times in PBS. Free-floating sections were then washed with PBS-T (0.3% Triton X-100), and blocked for 1 h in PBS-T containing 3% bovine serum albumin (BSA). Tissue was incubated overnight with goat polyclonal anti-DCC (A20, 1:500, Santa Cruz, RRID:AB_2245770). Sections were subsequently washed 3 × 10 mins in PBST with 3% BSA, incubated with donkey anti-goat Alexa-488 (1500; ThermoFisher Scientific, RRID: AB_143165), washed 2 X 10 mins with PBS, and incubated for 5 min with Hoechst stain (1:1000, Thermofisher Scientific, RRID: AB_2307445). Brain sections were then mounted on gelatin-coated glass microscope slides, cover-slipped using Prolong Fluoromount-G (ThermoFisher Scientific, RRID:SCR_015961). Immunofluorescent images were obtained using a Zeiss Axiovert (S100TV) microscope coupled with epifluorescent illumination at 10-20X (0.3–0.5 N. A.), captured using a CCD camera (Retiga R3, QImaging), and analyzed using Fiji software [16].

### Western blots

Animals were initially sedated with isoflurane gas and sacrificed via CO_2_ asphyxiation. Either whole-brain or microdissected CA1 and CA3 subregion hippocampal homogenates were collected for Western blot analysis. Tissue samples were homogenized in RIPA buffer supplemented with protease and phosphatase inhibitors (1 mg/mL aprotonin, 1 mg/mL leupeptin, 100 mM PMSF, and 0.5 M EDTA, 1 mM Na_3_VO_4_ and 1 mM NaF). Protein levels were quantified using the bicinchoninic acid protein assay (Pierce BCA kit, Thermo Fisher Scientific) and equal protein concentrations were loaded on acrylamide gels. Proteins were separated using SDS-PAGE electrophoresis on 10% polyacrylamide gels, electroblotted onto nitrocellulose membranes, blocked in 5% non-fat milk in PBS and incubated with primary antibodies overnight at 4 °C. The primary antibodies used were: goat polyclonal anti-DCC A20 (1:1000; Santa Cruz Biotechnology, RRID: AB_2245770), rabbit α-GAPDH (1:1000; Santa Cruz Biotechnology, RRID: AB_10167668), mouse anti-β-tubulin III (1:5000; Promega, RRID: AB_430874), rabbit α-phospho-S6 ribosomal protein (1:1000, Cell Signaling, RRID: AB_331682), mouse monoclonal α-PSD-95 (1:200, NeuroMAB, RRID: AB_2292909), mouse monoclonal α-Shank1 (1:200, NeuroMAB, RRID: AB_10673108), mouse monoclonal α-Shank2 (1:200, NeuroMAB, RRID: AB_2254586), mouse monoclonal α-Shank3 (1:200, NeuroMAB, RRID: AB_2187730), and rabbit α-p34-Arc/ARPC2 (1:1000, Millipore, RRID: AB_310447). Horseradish peroxidase (HRP) conjugated secondary antibody (1:10000) was subsequently applied and visualized by reaction with chemiluminesence reagent (Clarity Western Blotting Substrates Kit, BioRad) on radiography film (Carestream Blue X-ray film).

### Barnes maze

Spatial memory was evaluated in the Barnes maze as described previously [17], which consisted of a round table 70 cm in diameter with 12 equally spaced holes along the entire perimeter. One of the holes was designated as the target hole containing an underneath ramp attached to an escape tunnel. Training and test sessions lasted a total of 4 days [17, 18]. All objects and spatial cues in the room, along with the position of the experimenter, were unchanged during the training and testing sessions. The maze and escape tunnel were cleaned with 70% ethanol between trials to remove any olfactory cues. Mice were trained for 5–6 trials across 2–3 days, with each training trial 4 h apart per day. Mice were initially placed in the middle of the table within an opaque starting chamber for 20 s to randomize the direction they are facing. Once the chamber was lifted, a loud buzzer sound provided motivation to search for the escape tunnel. Each mouse was given 2 min to search for the target hole independently and allowed to stay in the box for 1 min. The buzzer sound was immediately stopped once the mouse entered the box. For all training sessions, number of nose pokes in the target holes, total number of holes searched, and latencies to the escape tunnel were counted by an experimenter blind to the genotype. Twenty-four hrs following the last training session, spatial memory was assessed using a probe trial in which the escape tunnel was removed. For quantitative analysis, the maze was divided into 4 quadrants with 3 holes per quadrants. Quadrants were labelled as: Target, Left, Right, and Opposite, with the target hole being the middle hole in the “Target” quadrant. The amount of time the mice spent in each quadrant was quantified by an experimenter blind to the genotypes.

### Novel object placement recognition

Mice were trained and tested using the novel object placement recognition (NOPR) test as a non-invasive measure of hippocampal-dependent spatial memory, which lasted a total of 3 days [8, 19, 20]. On Day 1, mice were habituated to the square testing chamber (50 cm X 36 cm, 26 cm-high wall) for 5 min without any added objects. On Day 2, during the “Sample Phase”, mice were exposed to two identical objects for 5 min in two separate training sessions that took place 4 h apart. Twenty-four hours following the last training session called the “Choice Phase”, one of the objects was moved to a novel location in the square chamber and the mice were provided 5 min to explore both objects. An overhead camera recorded the mice in the square chamber throughout training and testing, and exploration time was measured by an experimenter blind to the genotypes. Objects and the test chamber were cleaned with 70% ethanol between trials to remove olfactory cues.

Exploration times were calculated as the total time in the probe trial the animal investigated both objects (within one body length from an object with head pointed toward the object). Investigation ratios were calculated as the time spent exploring the novel placed object divided by the total time spent exploring both objects during the probe trial.

### Novel object recognition test

To assess for recognition memory, we employed the novel object recognition (NOR) test [21]. The test uses the same protocol as the NOPR test, but rather than moving one of the identical objects to a new location during the “Choice Phase”, one of the objects was replaced with a novel, non-identical object. The test was scored using the same parameters as the NOPR test: total exploration time and investigation ratio.

### Golgi staining and spine morphology

Mouse brains were processed (FD Rapid GolgiStain Kit; FD Neurotechnologies) and 100 μm sections cut with a cryostat. The length, width, and density of dendritic spines from apical dendrites of CA1 pyramidal neurons were traced and reconstructed, and analyzed using Fiji image software [16]. The type of spine (thin and mushroom) was also recorded.

### *Brain slice* in vitro *electrophysiology*

Acute transverse hippocampal brain slices were obtained from R4ag11-Cre /DCC^*fl/fl*^ (8–10 months old) and Grik4-Cre/DCC^*fl/fl*^ (8–10 months old) mice and age-matched control littermates (Cre-negative DCC^*fl/fl*^). Mice were deeply anaesthetized by intraperitoneal injection of a mixture of 2,2,2 – tribromoethyl alcohol and tert-amyl alcohol diluted at 2.5% in PBS, and transcardially perfused with ice-cold choline chloride-based solution containing (in mM): 110 choline-Cl, 1.25 NaH_2_PO_4_, 25 NaHCO_3_, 7 MgCl_2_, 0.5 CaCl_2_, 2.5 KCl, 7 glucose, 3 pyruvic acid, and 1.3 ascorbic acid, bubbled with carbogen (O_2_ 95%, CO_2_ 5%). The brain was quickly removed and 250 μm thick horizontal brain slices containing the hippocampus were cut using a vibrating microtome (VT1000s, Leica). Individual brain slices were allowed to recover for 1 h in artificial cerebrospinal fluid (ACSF) containing, in mM: 124 NaCl, 5 KCl, 1.25 NaH_2_PO_4_, 2 MgSO_4_, 26 NaHCO_3_, 2 CaCl_2_, and 10 glucose saturated with 95% O_2_ and 5% CO_2_ (pH ~ 7.3, 300 mOsm) at room temperature (22°-24 °C) prior to recordings.

Individual slices were positioned in a custom-built recording chamber, and continuously perfused with warmed (30 ± 2 °C) ACSF (TC324B, Warner Instruments). Current and voltage recordings of CA1 hippocampal pyramidal neurons were performed on an upright microscope (Nikon Eclipse or Scientifica SliceScope 2000) equipped with a micromanipulator (Sutter MP-225 or Scientifica Patchstar), a 40x or 60x water immersion objective (0.8 or 1.0 N.A., respectively), differential interference contrast optics, and coupled to a near-infrared charge-coupled device camera (NC70, MTI or SciCam, Scientifica). Borosilicate glass pipettes (Sutter Instruments) (tip resistance: 4–8 MΩ) were prepared using a horizontal puller (P-97, Sutter Instruments), and filled with an intracellular solution containing (in mM): 120 potassium gluconate, 20 KCl, 10 N-2-hydroxyethylpiperazine N′-2-ethanesulfonic acid (HEPES), 7 phosphocreatine diTris, 2 MgCl_2_, 0.2 ethylene glyco-bis (β-aminoethyl ether) N,N,N′,N′tetraacetic acid (EGTA), 4 Na^2 + −^ATP, 0.3 Na^+^-GTP (pH adjusted to 7.20–7.26 using KOH, 275–285 mOsm). Whole cell somatic recordings from visually-identified CA1 pyramidal neurons were performed in the presence of picrotoxin (100 μM) to block GABA_A_-mediated inhibitory synaptic transmission. Membrane potential and whole-cell current recordings were obtained using an Axopatch 200B or 700B amplifier (Molecular Devices). Current-clamp recordings were sampled at 20 kHz and filtered at 10 kHz, whereas voltage-clamp recordings were sampled at 10 kHz and filtered at 2 kHz. All data were acquired through an analog-to-digital converter (Digidata 1322A or 1550A, Molecular Devices) for storage on computer hard disk with pClamp software (v9.0 or v10.4, Molecular Devices).

Intrinsic electrophysiological characteristics of CA1 pyramidal neurons were assessed using a series of hyperpolarizing and depolarizing intracellular current pulses (− 200 pA to + 100 pA). Input resistance was calculated by measuring the peak voltage response to a − 100-pA current step (1000 ms). Rest membrane potential was measured 1 min following break-in to whole-cell configuration.

AMPAR-mediated synaptic currents were evoked using a bipolar platinum/iridium electrode (FHC, CE2C275) placed in the Schaffer collaterals ~ 200 μm from the recorded cell. During baseline periods prior to HFS, a pair of 0.1 ms bipolar current pulses (50 ms ISI) was delivered via a stimulus isolation unit (Isoflex, AMPI), and stimulus intensity was adjusted to evoke a response 65–75% of the maximal response at 200 μA. Input-output tests were conducted using increasing stimulus intensity from 0 to 200 μA in 25 μA increments. Changes in paired-pulse ratio were expressed as the peak amplitude of the second pulse as a function of the amplitude of the first pulse over a range of interstimulus intervals. Access and input resistances were continually monitored throughout the recording through a 50 ms, 5 mV voltage step 150 ms prior to synaptic stimulation, and data were discarded if series resistance changed > 20%.

Voltage-clamp recordings at − 70 mV were used to measure synaptic transmission at Schaffer collateral synapses. To assess HFS LTP in both DCC mutants and control littermates, the whole-cell recording mode was briefly changed to current-clamp, and LTP was induced using a single 1 s episode of 100 Hz stimulation, which has been previously demonstrated to elicit a long-lasting enhancement of evoked EPSCs in the adult hippocampus. Following stimulation, the cell was returned to voltage-clamp recording mode and held at − 70 mV to record evoked responses.

Spontaneous excitatory postsynaptic currents (sEPSCs) were recorded in voltage-clamp mode at a holding potential of − 70 mV in the presence of PTX (100 μM) to block GABA_A_-mediated synaptic currents. sEPSCs were analyzed using MiniAnalysis (Synaptosoft), and events were detected using a threshold of 7 pA (> 3 pA root mean square of baseline noise levels). Cumulative distribution plots were generated using an equal number of randomly-selected events per condition (100 per condition).

### Statistical analyses

Statistical analyses on parametric data were assessed using two-way repeated measures analysis of variance (ANOVA) followed by Bonferroni’s post-hoc test, one-way ANOVA followed by Tukey’s pairwise comparison test, and independent *t*-tests where appropriate. Analyses on nonparametric data were assessed using two-tailed Mann-Whitney test. Normality, homoscedasticity, and outlier tests were performed on all datasets. Data were analyzed using Clampfit 10.3 (Axon Instruments), MATLAB (Mathworks), Fiji [16], Photoshop (Adobe), MiniAnalysis (Synaptosoft), Prism 7 (Graphpad), and Sigmaplot 11 (Systat). Plotted data were then formatted in Adobe Illustrator CS6 (Adobe Systems).

## Results

### Subregion-specific conditional deletion of DCC in the adult hippocampus

DCC is enriched at synapses and widely expressed by neurons throughout the adult brain, including the hippocampus [9, 13]. To investigate whether DCC contributes to synaptic transmission in the adult brain through distinct pre- and post-synaptic mechanisms, we selectively deleted a floxed *dcc* allele from either CA3 or CA1 excitatory pyramidal neurons by Cre expression regulated by Grik4 (Grik4-Cre/DCC^*fl/fl*^) or R4ag11 (R4ag11-Cre/DCC^*fl/fl*^) promoters, respectively. Previous studies have demonstrated that Cre recombinase expression in Grik4-Cre and R4ag11-Cre mice is first detectable at P14 and P17, respectively, and recombination is maximal at ~ 8 weeks of age [11, 12, 15]. Critically, Cre expression is initiated after developmental axon guidance is complete. To confirm hippocampal subregion selectivity of DCC deletion, we examined the distribution of the Cre reporter, betagalactosidase (β-gal), in coronal brain sections from R4ag11-Cre/ROSA26 and Grik4-Cre/ROSA26 mice. In R4ag11-Cre/ROSA26 mice, β-gal expression was limited to the CA1 region of the hippocampus and dentate gyrus, but completely absent from the CA3 region (Supplemental Fig. 1A). Further, a substantially reduced level of DCC protein was detected in the CA1 region of the hippocampus of R4ag11-Cre/DCC^*fl/fl*^ mice (Supplemental Fig. 1B). Conversely, in Grik4-Cre/ROSA26 mice, β-gal expression was absent from the CA1 region and selectively expressed in CA3 (Supplemental Fig. 1C-D). These findings confirm subregion-specific deletion of DCC in the mature hippocampus using targeted Cre induced recombination.

### Selective deletion of DCC from CA1 glutamatergic neurons impairs spatial memory

In a previous study, DCC was demonstrated to co-fractionate with detergent-resistant components of postsynaptic density, and both confocal and immuno-electron microscopy supported post-synaptic enrichment of DCC, yet these findings did not rule out the possibility that DCC might be also be present on the presynaptic side of the Schaffer collateral synapse [13, 22]. DCC expression by glutamatergic excitatory neurons is required for spatial memory formation [13], however, in these studies DCC was deleted from both CA1 and CA3 hippocampal pyramidal neurons and the specific functional contributions made by presynaptic and postsynaptic DCC were not addressed.

**Fig. 1:**
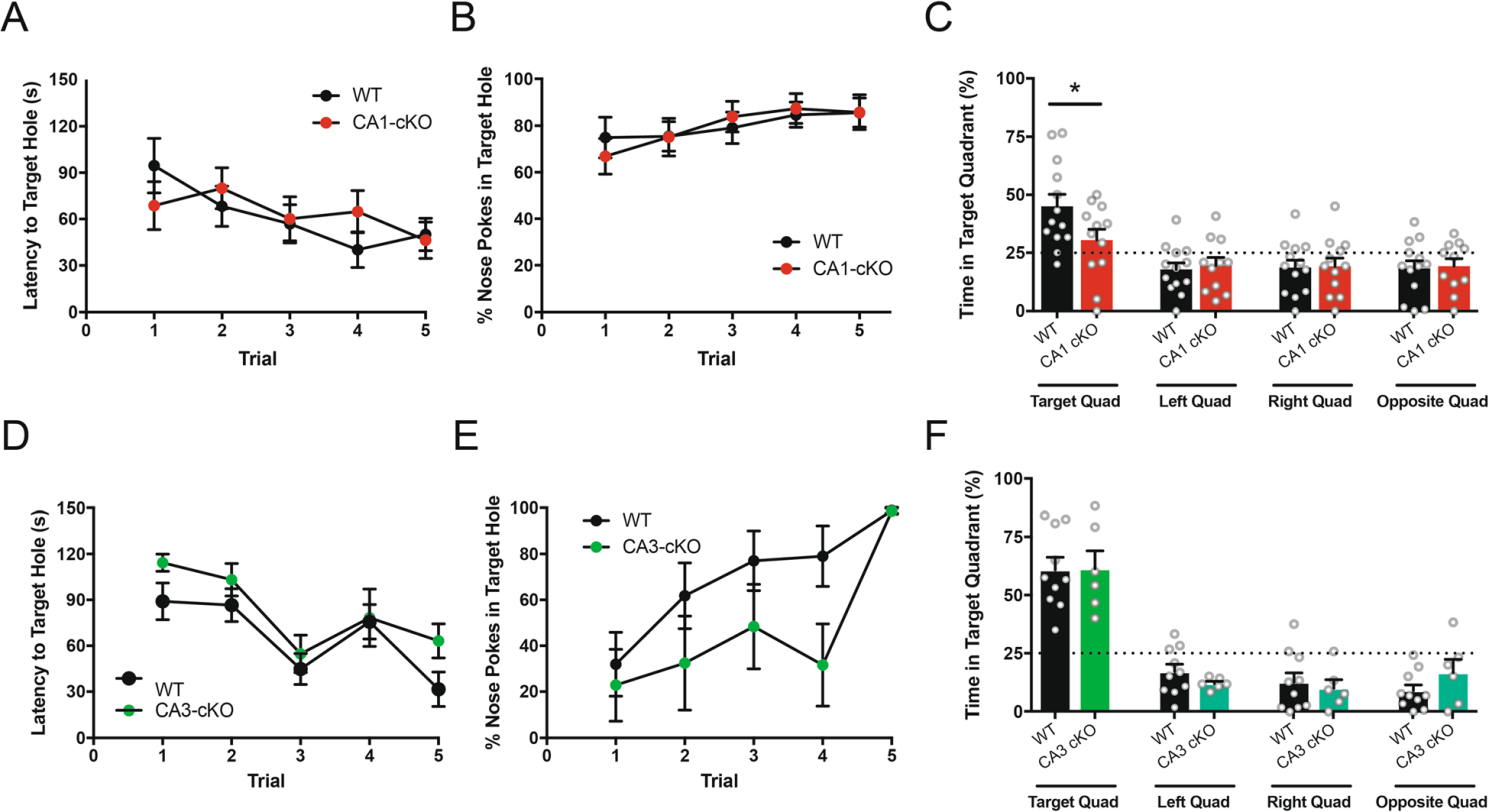
Conditional deletion of DCC in CA1 pyramidal hippocampal neurons but not CA3 pyramidal hippocampal neurons impairs spatial memory performance in Barnes maze test. **a**-**c** R4ag11-Cre/DCC^*fl/fl*^ (CA1 cKO) show no differences in latency to (**a**; Main effect of training: *F*_4,104_ = 2.54, *p* = 0.02; Interaction time by genotype: *F*_4,104_ = 1.10, *p* = 0.36) nor number of nose pokes in target hole (**b**; Main effect of training: *F*_4,200_ = 1.98, *p* = 0.044; Interaction time by genotype: *F*_4,200_ = 0.27, *p* = 0.89) during training phase, but spent significantly less time in the target hole quadrant compared to control littermates (black) (**C**; Interaction quadrant by genotype: *F*_1,46_ = 4.69, *p* = 0.03; Target quadrant, R4ag11-Cre/DCC^*f1/f1*^: 30.5 ± 4.7 s, Control: 45.0 ± 5.2 s; *p* = 0.007). Control, *n* = 13; CA1 cKO, *n* = 11. **d**-**f** Grik4-Cre/DCC^*fl/fl*^ (CA3 cKO) did not show any significant differences in escape latency (**d**; Main effect of training: *F*_5,70_ = 14.77, *p* < 0.001; Interaction time by genotype: *F*_5,70_ = 0.77, *p* = 0.57) or nose pokes into target hole (**e**; Main effect of training: *F*_4,56_ = 7.67, *p* < 0.001; Interaction time by genotype: *F*_4,56_ = 0.96, *p* = 0.43) during training, and did not differ from control mice in time spent in target quadrant (**f**; *t*_14_ = 0.05, *p* = 0.96). Control, *n* = 10; CA3 cKO, *n* = 6. * denotes *p* < 0.05 using Bonferroni posthoc tests. Barnes maze tests for R4ag11-Cre/DCC^*fl/fl*^ mice and Grik4-Cre/DCC^*fl/fl*^ mice, both on a C57Bl/6 J background, were run by different investigators which may contribute to differences in baseline performance

To assess the role of CA1 DCC expression in the consolidation of hippocampal-dependent spatial memory, R4ag11-Cre/DCC^*fl/fl*^ and wild-type age-matched littermate mice (Cre-negative DCC^*fl/fl*^ mice) were first tested using a modified Barnes maze paradigm. Briefly, mice were trained over 2 days on a circular open field table with equally spaced holes. One hole, the target, was attached to an escape box. The mouse seeking out the target relies on the innate preference of rodents to occupy enclosed spaces compared to open fields [17, 18]. R4ag11-Cre/DCC^*fl/fl*^ mice did not exhibit any difference in the latency to escape to (Fig. 1a), or number of nose pokes into (Fig. 1b), the target hole, compared to control mice, indicating intact sensory and motor functions, and consistent with normal cognitive spatial map formation [23]. Twenty-four hrs following the final training session, the mice were subjected to a probe trial in which the escape box was removed. R4ag11-Cre/DCC^*fl/fl*^ mice spent significantly less time in the target quadrant compared to control littermates, and explored each quadrant at chance levels (25%) (Fig. 1c). These findings provide evidence that deletion of DCC from CA1 pyramidal neurons results in a significant impairment in spatial memory performance.

Previous work has suggested functions for DCC on the axonal-presynaptic side of developing retinal ganglion cell (RGC) axons during target innervation [22], and although substantial evidence supports post-synaptic DCC localization and function [7, 13], a presynaptic function for DCC at the Schaffer collateral synapse had not been ruled out. To determine a role for DCC expression in CA3 pyramidal neurons during spatial memory, we tested the performance of Grik4-Cre/DCC^*fl/fl*^ mice and their control wild-type littermates using the same modified Barnes maze protocol. No significant differences were detected in escape latency (Fig. 1d), and Grik4-Cre/DCC^*fl/fl*^ mice showed a non-significant decrease in the number of nose pokes into the target hole (Fig. 1e) during the training phase (*p* = 0.055), however the relatively low number of Grik4-Cre/DCC^*fl/fl*^ mice tested (*n* = 6) may contribute to the lack of significance. Following a 24 h delay, mice were assessed for time spent in the quadrant previously containing the escape box. In contrast to selective deletion of DCC from CA1 pyramidal neurons, Grik4-Cre/DCC^*fl/fl*^ mice spent significantly more time in the target quadrant compared to other non-target quadrants, and did not differ significantly compared to wild-type littermates (Fig. 1f). These findings support the conclusion that selective deletion of DCC from CA3 pyramidal neurons does not impair spatial memory performance in the Barnes maze.

**Fig. 2:**
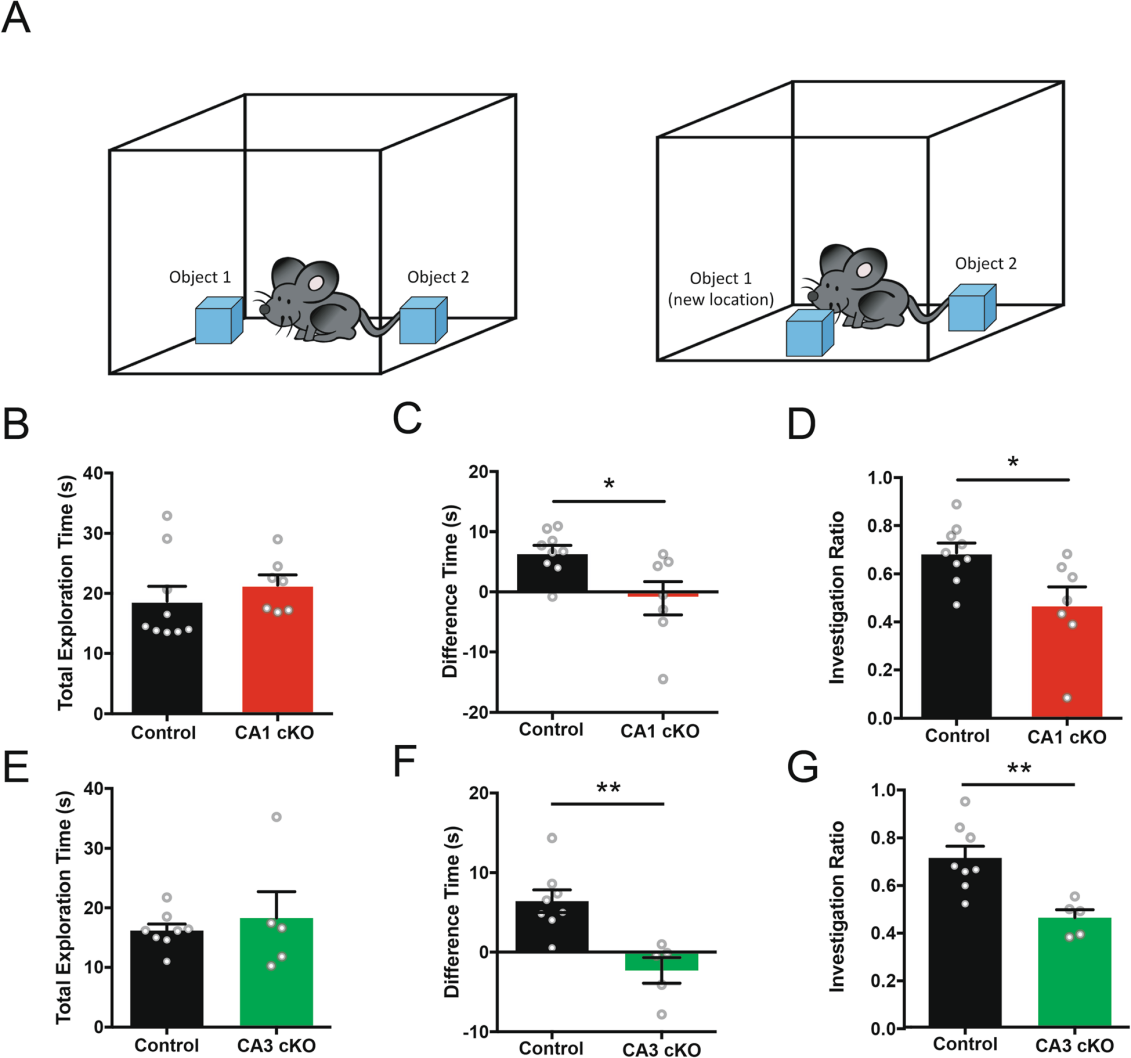
Conditional deletion of DCC from CA1 or CA3 pyramidal neurons impairs novel object place recognition. **a** Schematic representation of the novel object place recognition task. **b**-**d** R4ag11-Cre/DCC^*fl/fl*^ mice did not show significant differences in the total exploration time for either object (**b**; R4ag11-Cre/DCC^*fl/fl*^: *n* = 7, 21.4 ± 1.7 s,, Control: *n* = 9, 18.7 ± 2.5 s; *p* = 0.42), but spent significantly less time with the displaced object (**c**; R4ag11-Cre/DCC^*fl/fl*^: *n* = 7, − 1.0 ± 2.8 s,, Control: *n* = 9, 6.5 ± 1.2 s; *t*_14_ = 2.74, *p* = 0.016) and showed reduced investigation ratio (**d**; R4ag11-Cre/DCC^*fl/fl*^: *n* = 7, 0.47 ± 0.08, Control: *n* = 9, 0.69 ± 0.04; *t*_14_ = 2.69, *p* = 0.017). **e**-**g** Conditional deletion of DCC from CA3 pyramidal neurons resulted in no differences in total exploration time (E; Grik4-Cre/DCC^*fl/fl*^: *n* = 5, 18.3 ± 4.5 s, Control: *n* = 8, 16.2 ± 1.1 s, *p* = 0.59) compared to control mice (black), but significantly less time was spent with the displaced object (**f**; Grik4-Cre/DCC^*fl/fl*^: *n* = 5, − 2.3 ± 1.6 s, Control: *n* = 8, 6.4 ± 1.4 s; *t*_11_ = 3.93, *p* = 0.002) and the investigation ratio was significantly reduced (G; Grik4-Cre/DCC^*fl/fl*^: *n* = 5, 0.46 ± 0.03, Control: *n* = 8, 0.71 ± 0.05; *t*_11_ = 3.66, *p* = 0.003). * denotes *p* < 0.05, ** denotes *p* < 0.01

Impaired Barnes maze performance suggests a deficit in spatial memory function in mice lacking DCC expressed by CA1 hippocampal pyramidal neurons, however previous reports suggest that Barnes maze protocols may be anxiogenic [24]. To assess whether mice conditionally-lacking DCC in CA1 or CA3 pyramidal neuron showed impaired hippocampal-dependent spatial memory function using a non-anxiogenic task, we assessed R4ag11-Cre/DCC^*fl/fl*^ or Grik4-Cre/DCC^*fl/fl*^ mice using a novel object place recognition test (NOPR; Fig. 2a) [8, 20]. During the test phase, we observed no significant differences in total exploration time between R4ag11-Cre/DCC^*fl/fl*^ and control mice (Fig. 2b). However, consistent with impaired spatial memory, R4ag11-Cre/DCC^*fl/fl*^ mice spent significantly less time investigating the displaced object (Fig. 2c) and showed a significant reduction in investigation ratio compared to wild-type littermates (Fig. 2d). Grik4-Cre/DCC^*fl/fl*^ mice showed no difference in total exploration time (Fig. 2e), but spent less time with the displaced object (Fig. 2f) and decreased investigation ratio compared to control mice (Fig. 2g), indicating that DCC deletion from CA3 neurons impairs spatial memory performance on the NOPR task. These findings provide evidence that DCC expression by both CA1 and CA3 pyramidal neurons contribute to spatial memory formation that requires object discrimination.

**Fig. 3:**
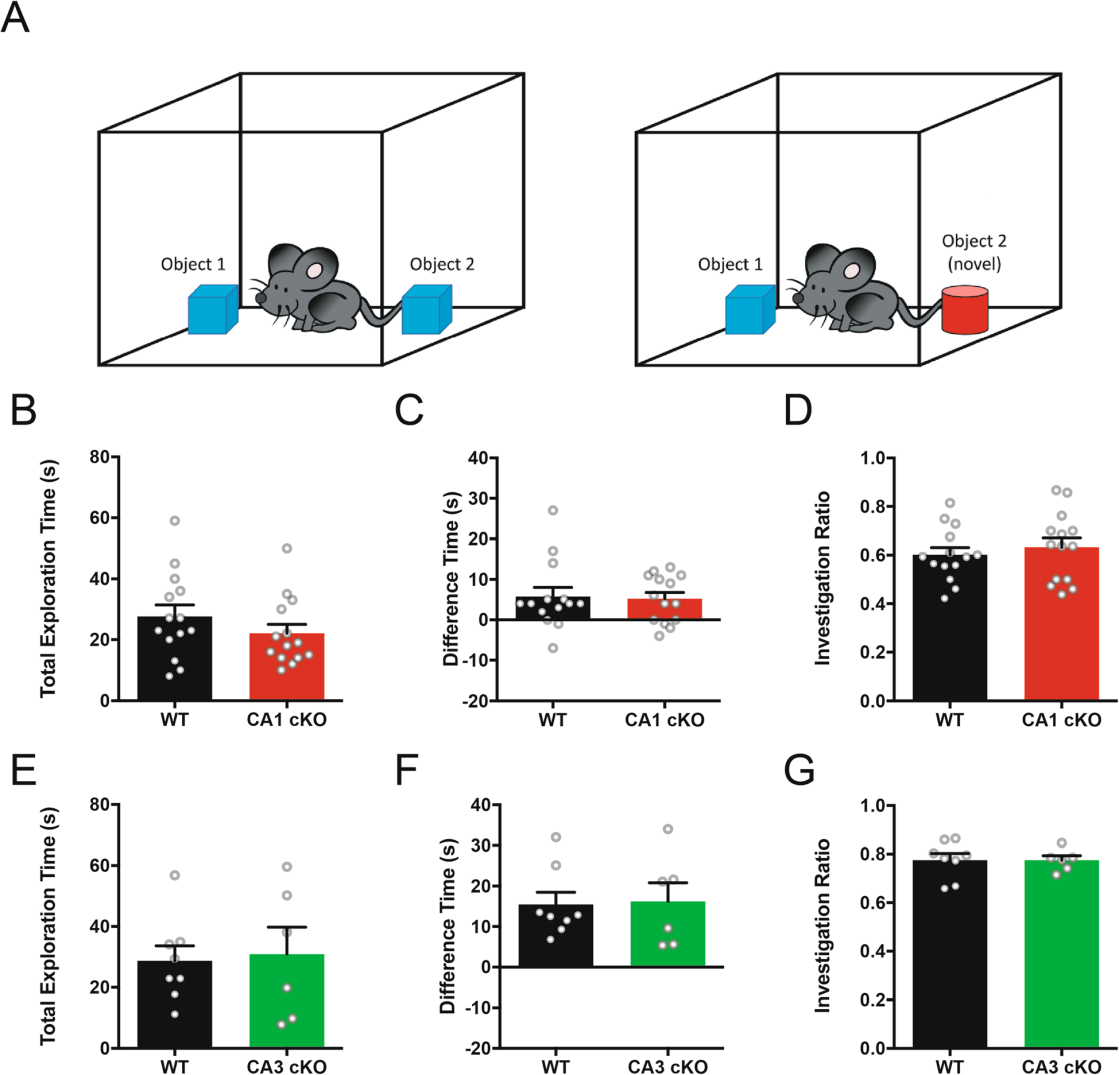
Conditional deletion of DCC from CA1 or CA3 pyramidal neurons does not impair performance of novel object recognition task. **a** Schematic representation of the novel object recognition task. **b-d** R4ag11-Cre/DCC^*fl/fl*^ (red) show no significant differences in total exploration time (**b**; R4ag11-Cre/DCC^*fl/fl*^: *n* = 14, 22.1 ± 3.0 s, Control: *n* = 14, 27.6 ± 3.7 s; *p* = 0.26), time spent with novel object (**c**; R4ag11-Cre/DCC^*fl/fl*^: *n* = 14, 5.2 ± 1.6 s, Control: *n* = 14, 5.8 ± 2.3 s; *p* = 0.83), or investigation ratio (**d**; R4ag11-Cre/DCC^*fl/fl*^: *n* = 14, 0.63 ± 0.4, Control: *n* = 14, 0.60 ± 0.03 s; *p* = 0.52) compared to control littermates (black). **e-g** Grik4-Cre/DCC^*fl/fl*^ did not spend more total time exploring objects (**e**; Grik4-Cre/DCC^*fl/fl*^: *n* = 6, 30.88 ± 8.851 s, Control: *n* = 8, 28.7 ± 4.9 s, *p* = 0.82), time spent with novel object (**f**; Grik4-Cre/DCC^*fl/fl*^: *n* = 6, 16.2 ± 4.6 s, Control: *n* = 8, 15.5 ± 3.0 s, *p* = 0.89), or investigation ratio (**g**; Grik4-Cre/DCC^*fl/fl*^: *n* = 6, 0.78 ± 0.02, Control: *n* = 8, 0.78 ± 0.02 s, *p* = 0.99)

Recognition of object location novelty evaluates the ability to recognize a familiar object in a new location, and co-opts intrinsic novelty-seeking behaviour [25]. However, NOPR requires visual discrimination and intact object recognition, which are mediated by non-hippocampal brain regions [26]. To examine whether non-hippocampal object recognition performance was impaired in mice conditionally-lacking DCC from either CA1 or CA3 pyramidal neurons, we assessed R4ag11-Cre/DCC^*fl/fl*^ or Grik4-Cre/DCC^*fl/fl*^ mice using a novel object recognition task (Fig. 3a). Consistent with normal visual function, we observed no significant differences in the total exploration time, proportion of time spent exploring novel objects, or investigation ratio in R4ag11-Cre/DCC^*fl/fl*^ (Fig. 3b-d) or Grik4-Cre/DCC^*fl/fl*^ (Fig. 3e-g) mice compared to control littermates. These findings indicate that mice lacking neuronal DCC expression in hippocampal subregions are not impaired in their ability to recognize novel objects.

### DCC deletion does not affect spiking activity in CA1 pyramidal neurons

**Fig. 4:**
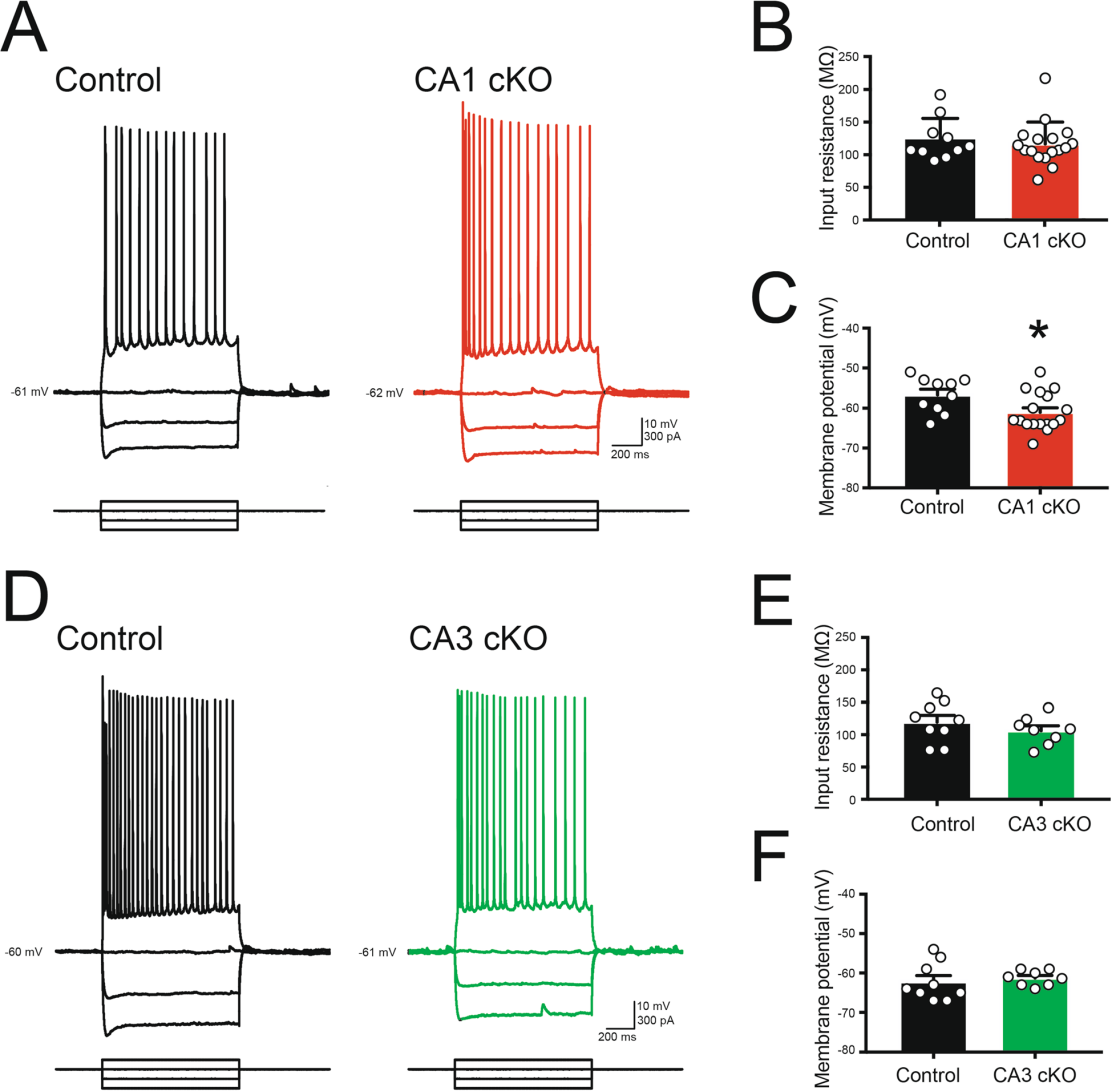
Intrinsic excitability of CA1 pyramidal neurons in R4ag11-Cre/DCC^*fl/fl*^ and Grik4-Cre/DCC^*fl/fl*^ mice. **a** Membrane potential responses to hyperpolarizing and depolarizing current pulses in CA1 pyramidal neurons derived from age-matched control (black, left) and R4ag11-Cre/DCC^*fl/fl*^ (CA1 cKO; red, right). **b-c** Group data showing resting membrane potential (**b**; R4ag11-Cre/DCC^*fl/fl*^: − 56.6 ± 1.5 mV, Control: − 61.1 ± 1.1 mV, *t*_25_ = 2.39, *p* = 0.024) and input resistance (**c**; R4ag11-Cre/DCC^*fl/fl*^: 116 ± 8 MΩ, Control: 123 ± 10 MΩ, *p* = 0.602) for CA1 pyramidal neurons from R4ag11-Cre/DCC^*fl/fl*^ and control mice. **d** Membrane potential traces in response to hyperpolarizing and depolarizing current pulses in CA1 pyramidal neurons from control (black, left) and Grik4-Cre/DCC^*fl/fl*^ (CA3 cKO; green, right). **e**-**f** Group data show no significant differences in input resistance (**e**; Grik4-Cre/DCC^*fl/fl*^: 119 ± 10 MΩ, Control: 106 ± 7 MΩ, *p* = 0.325) or resting membrane potential (**f**; Grik4-Cre/DCC^*fl/fl*^: − 61.3 ± 0.7 mV, Control: − 62.2 ± 1.6 mV, *p* = 0.62) between Grik4-Cre/DCC^*fl/fl*^ mice and control littermates. * denote *p* < 0.05

Changes in spatial memory function have been associated with alteration of overall neuronal excitability [27]. To assess whether conditional deletion of DCC alters intrinsic cellular excitability of CA1 pyramidal neurons, we recorded voltage responses to intracellular current injections using whole-cell patch clamp recordings from CA1 pyramidal neurons in acute hippocampal brain slices derived from 8 to 10 month old R4ag11-Cre/DCC^*fl/fl*^ or Grik4-Cre/DCC^*fl/fl*^ mice (Fig. 4a, d). Previous work provided evidence that DCC activates phospholipase C-γ (PLCγ) [13, 28], which in turn can modulate the open probability of various K^+^ channels through reduction of membrane-bound phosphatidylinositol 4,5-bisphosphate (PIP_2_) and alter neuronal input resistance [29]. Interestingly, we observed no change in steady-state input resistance (Fig. 4b), however we detected a slight hyperpolarization in the resting membrane potential of CA1 pyramidal neurons from R4ag11-Cre/DCC^fl/fl^ mice compared to control littermates (Fig. 4c). In contrast, no significant alteration of intrinsic cellular properties was detected in CA1 pyramidal neurons from Grik4-Cre/DCC^*fl/fl*^ mice (Fig. 4d-f).

### Effect of pre- and post-synaptic deletion of DCC on the Schaffer collateral synapse

**Fig. 5:**
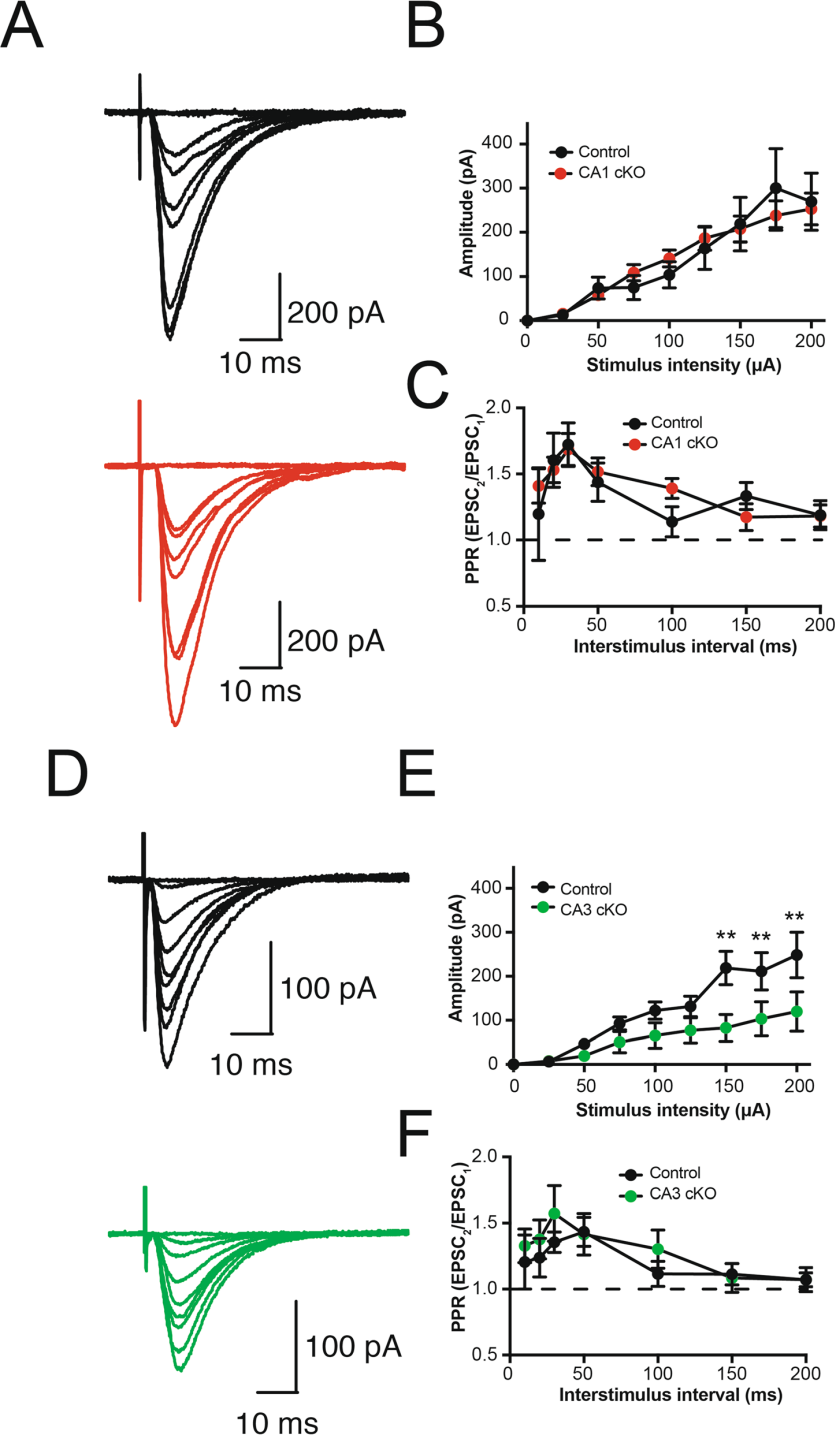
Selective deletion of DCC from CA3 pyramidal neurons impairs evoked synaptic transmission at Schaffer collateral synapses. **a** Representative traces of synaptic responses in CA1 pyramidal neurons in response to Schaffer collateral stimulation from control wild-type littermates (top, black) and R4ag11-Cre/DCC^*fl/fl*^ (CA1 cKO; bottom, red). **b**-**c** Group data show evoked EPSC amplitude in CA1 pyramidal neurons in response to incremental stimulation intensity of Schaffer collaterals (**b**; *F*_8,192_ = 0.18, *p* = 0.993) and paired-pulse ratio across a range of interstimulus intervals (**c**; ISI; Interaction: *F*_6,78_ = 0.76, *p* = 0.602) in R4ag11-Cre/DCC^*fl/fl*^ (CA1 cKO, red) and control littermates (black). **d**-**f** Representative traces (**d**) and group data (**e**; Interaction: *F*_8,112_ = 3.13, *p* = 0.003) from CA1 pyramidal neurons in control wild-type littermates (top, black) and Grik4-Cre/DCC^*fl/fl*^ (CA3 cKO; bottom, green) in response to incremental stimulation intensity of Schaffer collaterals. **f** Group data also show paired-pulse ratio across a range of ISI (Interaction: *F*_6,84_ = 0.53, *p* = 0.780). ** denotes *p* < 0.01

We have previously demonstrated that conditional deletion of DCC from principal excitatory neurons in the forebrain and hippocampus can strongly attenuate LTP at Schaffer collateral synapses in the adult hippocampus [13]. While substantial evidence indicates that DCC is present in postsynaptic dendritic spines, a role for presynaptic DCC had not been ruled out and it was not clear how pre- or post-synaptic DCC function might contribute to synaptic plasticity in the adult brain. To assess the effect of conditional deletion of DCC from either pre- or postsynaptic neurons in the hippocampus, we first recorded excitatory postsynaptic responses (EPSCs) from CA1 pyramidal neurons in response to stimulation of CA3 Schaffer collateral axons. Basal evoked EPSCs were measured in response to pulses presented with 25 μA increments in stimulation intensity up to 200 μA (Fig. 5a). No appreciable changes in amplitude of synaptic responses (Fig. 5b) or paired-pulse ratios (Fig. 5c; 10–200 ms ISI) were detected between R4ag11-Cre/DCC^*fl/fl*^ and wild-type controls. In contrast, the amplitude of evoked EPSCs in CA1 pyramidal neurons in response to Schaffer collateral stimulation was significantly lower at high stimulation intensities (150–200 μA) in Grik4-Cre/DCC^*fl/fl*^ mice compared to control littermates (Fig. 5d-e). These findings indicate that deletion of DCC from presynaptic CA3 neurons results in significantly decreased excitatory synaptic drive onto CA1 pyramidal neurons in the adult hippocampus, and suggest that DCC may influence the dynamics of presynaptic release.

DCC has been reported to associate with TRIM9, which can modulate soluble N-ethylmaleimide-sensitive factor-attachment protein (SNAP) receptor (SNARE)-mediated exocytosis mechanisms to regulate vesicular trafficking and neurotransmitter release [30, 31]. To determine whether conditional deletion of DCC was associated with changes in presynaptic release dynamics, we assessed the paired pulse ratio across a range of interstimulus intervals (10–200 ms ISI) in Grik4-Cre/DCC^*fl/fl*^ mice and control littermates. No change in PPR was detected across all ISIs in Grik4-Cre/DCC^*fl/fl*^, consistent with a lack of effect of either pre- or postsynaptic DCC deletion on the probability of presynaptic vesicular release (Fig. 5f).

**Fig. 6:**
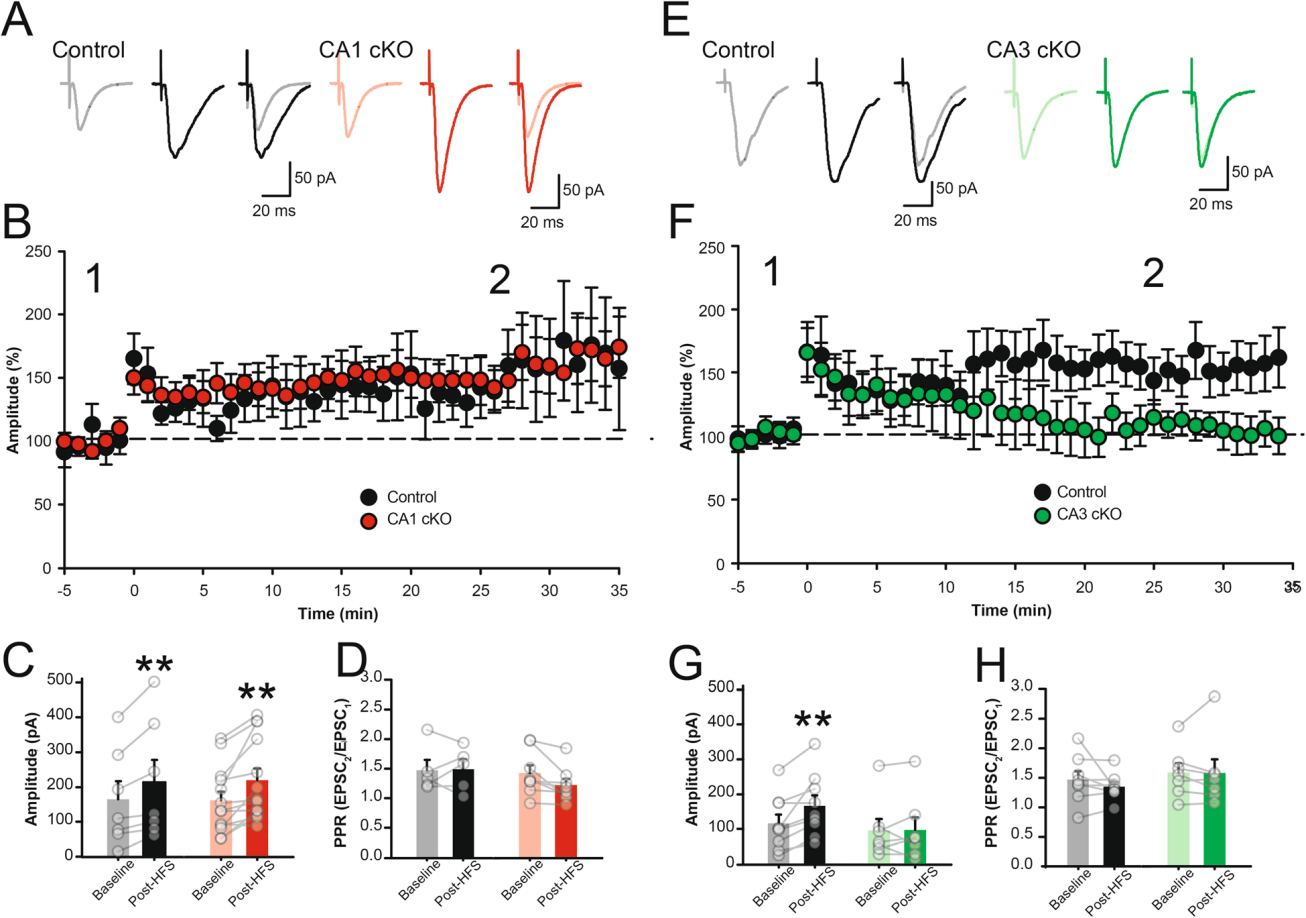
Deletion of DCC from CA3 pyramidal neurons, but not CA1 pyramidal neurons, impairs HFS-induced LTP in the adult hippocampus. **a**-**b** Representative evoked AMPAR-mediated EPSCs (**a**) and group data (**b**) recorded at − 70 mV in response to Schaffer collateral stimulation from control littermates (left, black) and R4ag11-Cre/DCC^*fl/fl*^ (CA1 cKO; right, red) prior to and following (20 min post-HFS) brief high-frequency stimulation (1 s at 100 Hz). The amplitude of EPSCs remained significantly potentiated after 25 min in slices from both R4ag11-Cre/DCC^*fl/fl*^ (144 ± 10% of baseline, *p* < 0.001) and controls (140 ± 12% of baseline, *p* = 0.02) compared to baseline values (Main effect of HFS: *F*_1,18_ = 20.78, *p* < 0.001; Interaction genotype X HFS: *F*_1,18_ = 0.20, *p* = 0.659). **c** Paired-pulse ratio (50 ms ISI) was not significantly changed between R4ag11-Cre/DCC^*fl/fl*^ (CA1 cKO) mice and control littermates (*p* = 0.602). **d**-**f** Representative evoked AMPAR-mediated EPSCs (**d**) and group data (**e**) recorded at − 70 mV in response to Schaffer collateral stimulation from control littermates (left, black) and Grik4-Cre/DCC^*fl/fl*^ (CA3 cKO; right, green) prior to and following (20 min post-HFS) brief high-frequency stimulation (1 s at 100 Hz). AMPAR-mediated EPSCs were not significantly different from baseline values (113 ± 6% of baseline values, *p* = 0.913) compared to controls (145 ± 9% of baseline, *p* = 0.002) (Interaction for genotype X HFS: *F*_1,14_ = 6.30, *p* = 0.025), with no appreciable change in the paired-pulse ratio (**f**; Interaction for genotype X HFS: *p* = 0.780). ** denotes *p* < 0.01

Our previously reported deletion of DCC from CA3 and CA1 pyramidal neurons [13], or the current selective conditional deletion from either CA1 pyramidal neurons (R4ag11-Cre/DCC^*fl/fl*^) or CA3 pyramidal neurons (Grik4-Cre/DCC^*fl/fl*^), in all cases is sufficient to impair spatial memory performance (Fig. 2). Spatial memory performance requires de novo formation of location-specific neuronal firing fields (place fields), and place cell firing patterns can induce long-lasting NMDAR-dependent changes in the strength of synaptic connections, suggesting that deletion of DCC may disrupt synaptic potentiation in response to high-frequency stimulation [32]. To determine if postsynaptic deletion of DCC impairs LTP, we recorded evoked EPSCs in CA1 pyramidal neurons from R4ag11-Cre/DCC^*fl/fl*^ mice in response to Schaffer collateral stimulation in the presence of the GABA_A_ antagonist, picrotoxin (100 μM) (Fig. 6a). Brief high-frequency stimulation (100 Hz for 1 s) resulted in significant short-term facilitation of EPSCs in both R4ag11-Cre/DCC^*fl/fl*^ mice and control littermates. Surprisingly, the amplitude of EPSCs remained significantly potentiated after 25 min in slices from both R4ag11-Cre/DCC^*fl/fl*^ (144 ± 10% of baseline, *p* < 0.001) and controls (140 ± 12% of baseline, *p* = 0.02) compared to baseline values (Fig. 6b-c). Furthermore, no change in paired-pulse ratio occurred (Fig. 6d). These findings demonstrate that conditional postsynaptic deletion of DCC from CA1 pyramidal neurons does not impact NMDAR-dependent LTP at Schaffer collateral synapses.

Although mediated through changes in the postsynaptic neuron, LTP may also involve alteration of presynaptic function, and homeostatic alteration of release dynamics may contribute to Hebbian synaptic plasticity [33, 34]. To determine whether deletion of DCC from CA3 pyramidal neurons might impair HFS-induced LTP at Schaffer collateral synapses, we recorded evoked EPSCs in CA1 pyramidal neurons derived from Grik4-Cre/DCC^*fl/fl*^ mice in response to Schaffer collateral stimulation (Fig. 6e). A brief high-frequency tetanus (100 Hz for 1 s) resulted in an immediate potentiation of synaptic responses in CA1 pyramidal neurons. However, by 25 mins after HFS, synaptic responses in slices from Grik4-Cre/DCC^*fl/fl*^ returned to baseline values (113 ± 6% of baseline values, *p* = 0.913) compared to controls (145 ± 9% of baseline, *p* = 0.002) (Fig. 6f-g). We observed no changes in paired-pulse ratio following HFS in brain slices from either Grik4-Cre/DCC^*fl/fl*^ mice or their control littermates (Fig. 6h). Together, these results indicate that deletion of presynaptic, but not postsynaptic, DCC impairs HFS-induced LTP in the Schaffer collateral pathway of the adult hippocampus.

### Postsynaptic deletion of DCC reduces basal synapse strength

**Fig. 7:**
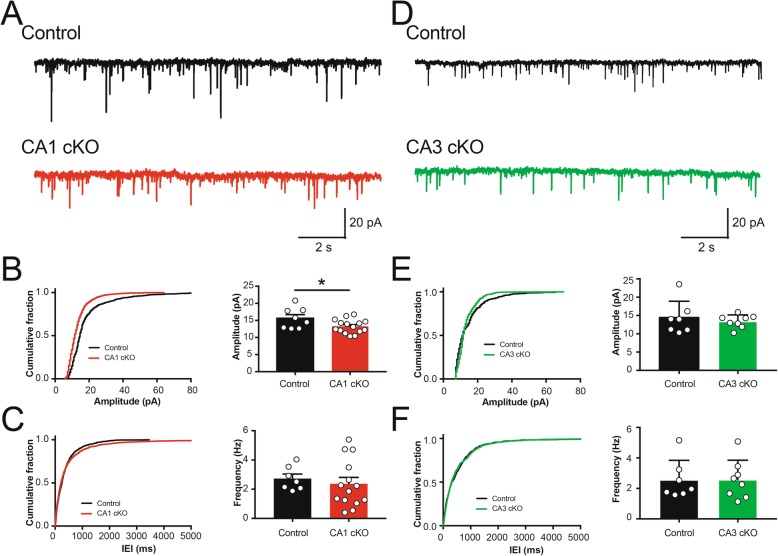
Conditional deletion of DCC from CA1 pyramidal neurons decreases sEPSC amplitude. **a** Representative spontaneous excitatory postsynaptic current (sEPSC) traces in CA1 pyramidal neurons from control (black, top) and R4ag11-Cre/DCC^*fl/fl*^ (CA1 cKO; red, bottom) hippocampal slices. **b**-**c** Cumulative distribution plots (left) and group data (right) show a significant decrease in the average amplitude of sEPSCs in R4ag11-Cre/DCC^*fl/fl*^ mice (**b**; R4ag11-Cre/DCC^*fl/fl*^: *n* = 15, 13.2 ± 0.5 pA, Control: *n* = 8, 15.6 ± 1.0 pA; *t*_21_ = 2.26, *p* = 0.034) but no change in average frequency (**c**; R4ag11-Cre/DCC^*fl/fl*^: *n* = 15, 2.3 ± 0.4 Hz, Control: *n* = 8, 2.7 ± 0.3 Hz; *p* = 0.59). **d** Representative sEPSC traces from CA1 pyramidal neurons from control (black, top) and Grik4-Cre/DCC^*fl/fl*^ mice (CA3 cKO; green, bottom). **e**-**f** Cumulative distribution plots (left) and group data (right) show no changes in average sEPSC amplitude (**e**; Grik4-Cre/DCC^*fl/fl*^: *n* = 8, 13.3 ± 0.6 pA, Control: *n* = 7, 14.3 ± 1.7 pA; *p* = 0.60) or average frequency (**f**; Grik4-Cre/DCC^*fl/fl*^: *n* = 8, 2.6 ± 0.5 Hz, Control: *n* = 7, 2.6 ± 0.5 Hz; *p* = 0.982) between Grik4-Cre/DCC^*fl/fl*^ and control littermates. K-S test for cumulative distribution plots. * denotes *p* < 0.05

While no differences in evoked synaptic strength were observed in R4ag11-Cre/DCC^*fl/fl*^ mice, we postulated that individual excitatory synapses may show reduced sensitivity to presynaptic transmitter release. To assess basal synaptic strength in R4ag11-Cre/DCC^*fl/fl*^ mice, we recorded AMPAR-mediated spontaneous EPSCs (sEPSCs) in the presence of picrotoxin (100 μM) (Fig. 7a). We found that the amplitude of sEPSC events was significantly decreased compared to events from age-matched control mice (Fig. 7b). In contrast, we detected no significant differences in sEPSC frequency (Fig. 7c). These findings indicate that deletion of DCC from CA1 pyramidal neurons impairs the relative strength of individual synapses, without affecting the overall number of synaptic inputs.

Presynaptic deletion of DCC prevented expression of HFS-induced LTP (Fig. 6), however it remained unclear whether this resulted in alteration of network-level synaptic properties. To examine whether deletion of DCC in CA3 pyramidal neurons affected basal spontaneous synaptic transmission, we recorded sEPSCs in CA1 pyramidal neurons from Grik4-Cre/DCC^*fl/fl*^ mice in the presence of picrotoxin (100 μM) (Fig. 7d). We detected no difference in average sEPSC amplitude or frequency between genotypes (Fig. 7e-f), indicating that deletion of DCC from CA3 does not impair basal network-level synaptic inputs onto CA1 pyramidal neurons.

### Deletion of postsynaptic DCC alters dendritic spine morphology

**Fig. 8:**
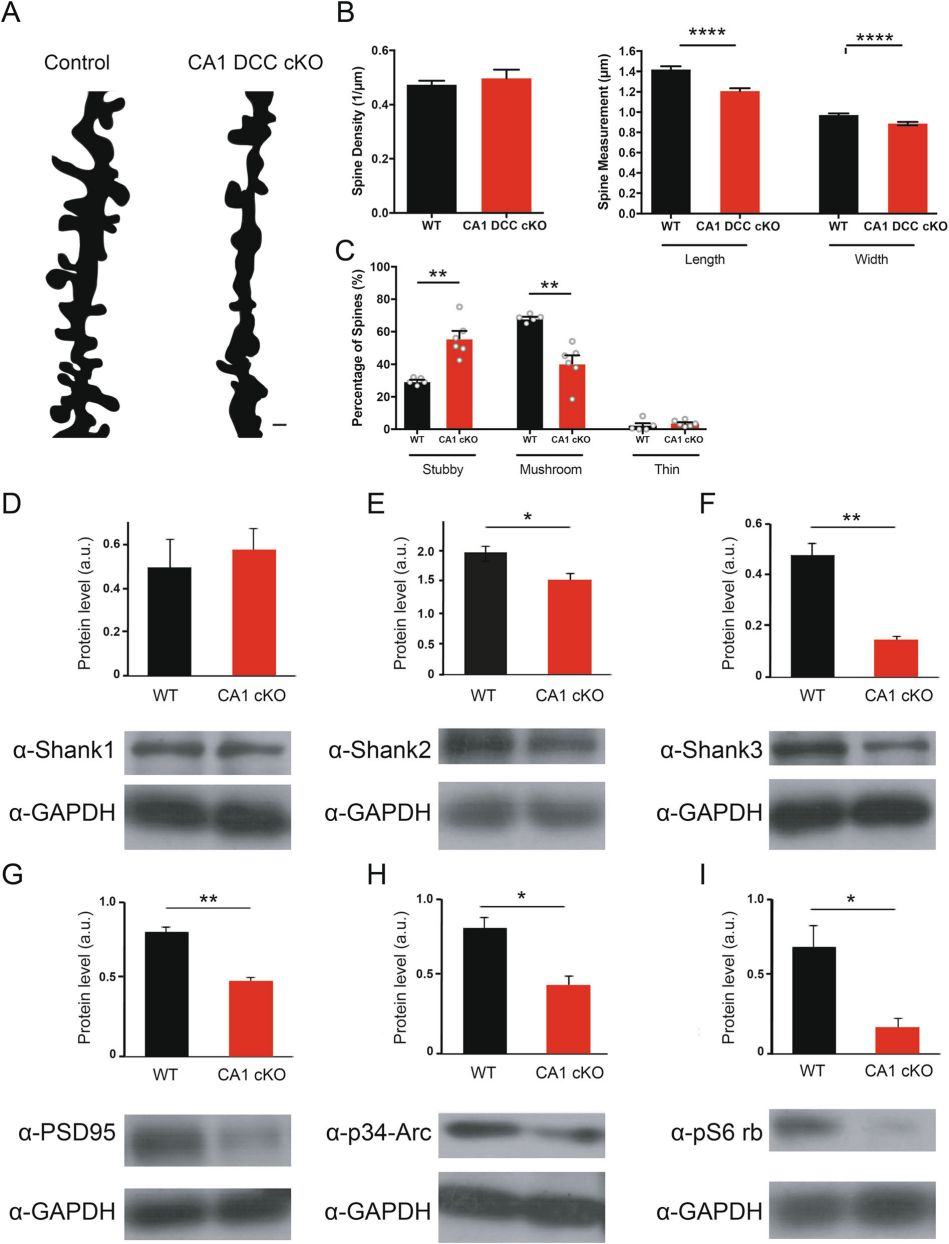
Alterations in dendritic spine morphology in CA1 pyramidal neurons from R4ag11-Cre/DCC^*fl/fl*^ mice. **a** Representative camera lucida reconstructions of CA1 pyramidal neuron apical dendrites from control (left) and R4ag11-Cre/DCC^*fl/fl*^ mice. Scale bar = 1 μm. **b** Group data show no significant difference in average spine density between control and R4ag11-Cre/DCC^*fl/fl*^ mice (left), but significant reductions in average spine length and spine head width (right). **c** Dendritic spine type classification reveals a significant increase in number of stubby-type spines (R4ag11-Cre/DCC^*fl/fl*^: 55.8 ± 4.6%, Control: 29.6 ± 0.9%; *t*_5.4_ = 5.59, *p* = 0.002) and significant decrease in the number of mushroom-type dendritic spines (R4ag11-Cre/DCC^*fl/fl*^: 40.6 ± 4.9%, Control: 68.2 ± 1.0%; *t*_5.47_ = 5.40, *p* = 0.002), with no detectable differences in thin-type spines (*p* = 0.41). **d**-**i** Group data (top) and representative Western blots from hippocampal homogenates of control and R4ag11-Cre/DCC^*fl/fl*^ mice showing levels of SHANK1 (**d**), SHANK2 (**e**), SHANK3 (**f**), PSD-95 (**g**), p34-Arc (**h**), and phosphorylated S6 ribosomal protein (**i**). *: *p* < 0.05; **: *p* < 0.01. Data are presented as mean ± SEM

Synaptic modification during learning is thought to be critical for the consolidation of new memories [1]. We found that animals lacking DCC in CA1 pyramidal neurons exhibited significant deficits in spatial memory, but did not exhibit any change in the propensity for CA1 LTP induction (Figs. 1 and 6). However, we also identified smaller sEPSC amplitudes in the R4ag11-Cre/DCC^*fl/fl*^ mice, suggesting a relatively subtle change in excitatory synaptic transmission in the absence of DCC, possibly in postsynaptic function, which may underlie spatial memory impairment. Indeed, spatial memory function is associated with changes in dendritic spine morphology in CA1 pyramidal neurons [35–37], and our previous conditional deletion of DCC from both CA1 and CA3 pyramidal neurons in T29–1 *CamKIIα-Cre*/DCC^*fl/fl*^ mice resulted in an increase in stubby-type dendritic spines along the apical dendrites of CA1 pyramidal neurons [13]. We therefore quantified dendritic spine density and morphology in CA1 pyramidal neurons from R4ag11-Cre/DCC^*fl/fl*^ and control littermates using the Golgi-Cox staining method (Fig. 8a). Dendritic spine density was unchanged between R4ag11-Cre/DCC^*fl/fl*^ and control littermates (Fig. 8b). In contrast, the average length and width of dendritic spines were significantly reduced along the apical dendrites of CA1 pyramidal neurons from R4ag11-Cre/DCC^*fl/fl*^ mice compared to control littermates (Fig. 8b). These results indicate that postsynaptic deletion of DCC from CA1 pyramidal neurons does not affect dendritic spine density, but reduces average spine length and width along the apical dendrites of CA1 pyramidal neurons.

Dendritic spines can be grouped into three major categories: thin, mushroom, and stubby [38]. Thin-type spines have longer lengths than head diameters, and mushroom-type spines have head sizes that greatly exceed neck length [39]. Stubby-type spines are transient, characterized by short neck lengths and wide diameters, and are considered immature, showing robust increases in overall volume following plasticity-inducing protocols [40]. To determine whether DCC deletion in CA1 pyramidal neurons impacted dendritic spine morphology, we examined the density of different spine types (mushroom, thin, stubby) in wild-type littermates and R4ag11-Cre/DCC^*fl/fl*^ mice. We detected a significant increase in the number of stubby spines and reduction of mushroom spines in R4ag11-Cre/DCC^*fl/fl*^ mice, with no significant change in thin spines (Fig. 8c). Together, these findings identify a DCC-mediated contribution to the maturation, maintenance, and stabilization of mature spine structures in CA1 pyramidal neurons.

Dendritic spines are the primary site of excitatory synapses in the mammalian brain, and are associated with synaptic scaffolding proteins such as SH3 and multiple ankyrin repeat domains proteins (SHANKs) [41]. Due to the observed alterations in dendritic spine maturation and stability, we hypothesized that postsynaptic deletion of DCC may reduce relative levels of scaffolding proteins. To assess whether postsynaptic deletion of DCC alters SHANK expression, we measured SHANK1, SHANK2, and SHANK3 levels in hippocampal homogenates from wildtype and R4ag11-Cre/DCC^*fl/fl*^ mice. Interestingly, SHANK2 and SHANK3 protein levels were significantly reduced in R4ag11-Cre/DCC^*fl/fl*^ mice compared to wildtype controls, but no difference was detected in levels of SHANK1 (Fig. 8d-f).

SHANKs interact with a number of cytoplasmic and membrane-bound synaptic proteins, including postsynaptic density 95 (PSD-95) [42]. Loss of DCC from CA1 pyramidal neurons resulted in reduced levels of PSD-95 protein (Fig. 8g). Additionally, we detected reduced amounts of the actin-regulating protein p34-Arc, a component of the Arp2/3 complex that initiates F-actin branch formation (Fig. 8h). These findings indicate that DCC plays a critical role governing the levels of postsynaptic scaffolding and cytoskeletal regulatory proteins.

Previous studies have provided evidence that DCC regulates protein translation at synapses and colocalizes with translational machinery and newly synthesized proteins [43]. To investigate a possible contribution of DCC to the regulation of protein translation, we assessed levels of phosphorylated S6 ribosomal protein in hippocampal homogenates from R4ag11-Cre/DCC^*fl/fl*^ and age-matched wildtype controls. Interestingly, levels of phosphorylated S6 ribosomal protein were significantly reduced in tissue from R4ag11-Cre/DCC^*fl/fl*^ mice compared to age-matched wildtype controls, suggesting that DCC may regulate protein translation in CA1 neurons in the mature hippocampus (Fig. 8i).

## Discussion

Recent evidence has demonstrated an emerging role for axon guidance proteins in the adult brain, however little is known regarding the subcellular locus of action of these molecules [6, 44–46]. Here, we show that pre- or post-synaptic deletion of the netrin-1 receptor DCC from excitatory glutamatergic neurons in the mouse hippocampus reveals complementary roles in spatial memory, synaptic transmission, and synaptic plasticity. We demonstrate that conditional deletion of DCC from CA1 pyramidal neurons significantly impaired spatial memory tasks, reduced sEPSC amplitude, altered dendritic spine morphology, and reduced levels of SHANK2, SHANK3, PSD-95, p34-Arc, and phosphorylated S6 ribosomal proteins compared to control wild-type littermates, but did not block HFS-induced LTP at Schaffer collateral synapses. Conditional deletion of DCC from CA3 pyramidal neurons selectively impaired spatial memory performance on the NOPR task, and resulted in a striking attenuation of HFS-induced LTP. These findings identify distinct roles for DCC distributed on either side of the Schaffer collateral synapse in spatial information processing, as well as functional and structural synaptic plasticity in the adult hippocampus. More specifically, postsynaptic deletion of DCC fails to impair basal synaptic transmission, and does not block synaptic capacity for classic activity-dependent plasticity paradigms. Rather, our findings provide evidence that postsynaptic DCC may govern long-term stability of synapses through effects on protein synthesis, recruitment of synaptic scaffolding proteins, and synapse maturation without impacting the overall density of excitatory synaptic connections. Conversely, deletion of DCC from CA3 pyramidal neurons resulted in reduced basal synaptic transmission and disrupted LTP expression in CA1 neurons, suggesting that DCC in CA3 pyramidal neurons may regulate mechanisms of vesicular mobilization and/or fusion at the Schaffer collateral synapse. Interestingly, deletion of DCC from CA3 pyramidal neurons did not impact the amplitude or frequency of spontaneous excitatory postsynaptic currents recorded in CA1 pyramidal neurons, which rely on stochastic mobilization and fusion, suggesting that the mechanisms underlying vesicular fusion itself are not compromised in Grik4-Cre/DCC^*fl/fl*^ mice. However, sEPSCs in CA1 pyramidal neurons are driven by excitatory inputs from both CA3 pyramidal neurons as well as extrahippocampal cortical neurons, which may mask presynaptic changes in sEPSC amplitude at Schaffer collateral synapses in CA3 DCC cKO mice. Together, these findings suggest that presynaptic DCC regulates large-scale mobilization of glutamate-containing neurotransmitter vesicles, although the specific mechanism underlying this regulation requires further investigation.

### Postsynaptic deletion of DCC may regulate late-phase synaptic changes following memory formation

Deletion of DCC expressed by CA1 pyramidal neurons did not affect overall spine density, yet increased the proportion of stubby-type spines and decreased mushroom-type spines. Stubby-type spines predominate during early postnatal life, and are thought to represent an early stage of synaptogenesis [47]. During the second postnatal week in mice, the majority of synapses formed are stubby-type protrusions from dendritic shafts, which in turn decrease in number by adulthood, when ~ 80% of dendritic spines have a mushroom-type structure [48]. During early development, DCC and netrin-1 promote glutamatergic synaptogenesis, directing the accumulation of presynaptic and postsynaptic proteins such as synaptophysin and PSD-95, respectively [49]. Cre expression in R4ag11-Cre mice starts at ~P17, following completion of axon guidance but during the process of synapse maturation [11, 39]. Postsynaptic loss of DCC in CA1 pyramidal neurons may impair the maturation or maintenance of more stable mushroom spines, and thereby limit the accumulation of PSD-95 and other membrane-associated guanylate kinases-related proteins such as SAP97, which are necessary for netrin-1 mediated potentiation of hippocampal synapses [7, 49]. As a result, the lack of DCC in CA1 pyramidal neurons may arrest spine maturation and attenuate ongoing basal synaptic transmission in adulthood, reducing the average strength of EPSCs in adult CA1 pyramidal neurons.

Dendritic spine shape and size can regulate synaptic transmission through modulation of electrical resistance [50]. Stubby-type spines are considered relatively transient, with only ~ 17% of all stubby spines in the hippocampus persisting following a 24 h period, and contain fewer postsynaptic proteins compared to more mature spine types [51]. Consistent with a role in learning and memory, spatial training decreased the number of stubby spines and increased the number of mushroom-type spines along the apical dendrites of CA1 pyramidal neurons, suggesting that stubby spines mature into more stable mushroom-type spines following consolidation [52]. Selective deletion of DCC from CA1 pyramidal neurons may therefore allow for normal spatial behaviour but impair memory consolidation due to impaired maturation and stabilization of immature synapses. A preponderance of relatively immature PSDs in transient dendritic spines may contribute to altered basal spontaneous synaptic amplitude and reduced PSD-95 in animals lacking DCC in CA1 pyramidal neurons (R4ag11-Cre/DCC^*fl/fl*^) (Figs. 7, 8 g). However, the persistence of LTP in R4ag11-Cre/DCC^*fl/fl*^ mice (Fig. 6) suggests that the increased proportion of stubby spines is sufficient to support at least the early phases of activity-dependent synaptic potentiation.

Consistent with impaired long-term memory consolidation, we observed behavioural deficits in spatial tasks that required > 24 h delay periods between training and test probes. Although not assessed here, it is possible that the HFS-induced LTP produced in R4ag11-Cre/DCC^*fl/fl*^ mice may not exhibit the subsequent protein synthesis-dependent LTP late phase [53]. Recent work using *Aplysia* neurons demonstrated that local mRNA translation at synaptic sites can be initiated by netrin-1, suggesting that DCC may regulate protein synthesis following early phases of plasticity [54]. We have previously demonstrated that bath application of netrin-1 rapidly recruits synaptic GluA1-containing AMPARs through a DCC-dependent mechanism [7], but the contribution of netrin-1 to late phase LTP in the mammalian brain is not known. Consistent with playing multiple key roles in different phases of LTP, initial increases in Ca^2+^-permeable AMPARs facilitate rapid synaptic strengthening, which can be followed by GluA1-containing endocytosis and protein synthesis-dependent synaptic stabilization through the insertion of GluA2-containing Ca^2+^-impermeable receptors [3, 55–57].

### Role of DCC in synaptic changes associated with memory consolidation

Netrin-1 is released at synaptic and perisynaptic sites in response to NMDAR activation to facilitate GluA1-containing AMPAR insertion through a DCC-dependent signaling cascade, but it remains unclear how DCC deletion may affect netrin-1 levels or release [7, 46]. Additionally, a number of ligands have been identified for DCC, including draxins and cerebellins, and netrin-1 binds a number of different receptors, including DSCAM, APP, UNC5, and Neogenin [6]. Although not assessed here, it will be imperative in future studies to investigate the possible differential expression of other ligands and receptors in animals that lack DCC.

While high-frequency tetanus-induced LTP has been considered as an experimental model of the neuronal changes that underlie learning and memory, recent studies provide evidence for possible functional dissociations between this form of synaptic plasticity and behavioural performance on spatial memory tasks. Indeed, memory deficits in the Morris Water Maze can be associated with no appreciable change in LTP induction or expression [58, 59]. Here, we identified a significant deficit in Barnes maze spatial performance in R4ag11-Cre/DCC^*fl/fl*^ mice, with no change in LTP induction at the Schaffer collateral synapse in vitro, whereas animals lacking DCC in CA3 neurons exhibited no deficit in Barnes maze performance, yet did show impaired LTP. These findings highlight that synaptic plasticity at Schaffer collateral synapses observed in vitro is not necessarily congruent with spatial memory consolidation, and suggest that other forms of synaptic plasticity, or other synaptic loci, may more accurately reflect the synaptic processes underlying memory formation.

While failing to reach statistical significance, we note a trend towards impaired learning in Grik4-Cre/DCC^*fl/fl*^ mice compared to control littermates. These results suggest that deletion of DCC from CA3 pyramidal neurons may impair synaptic mechanisms that underlie learning processes, but do not contribute to retrieval of stored spatial memory. This is consistent with previous work showing a critical role for CA3 neurons in new spatial learning, but not consolidation or subsequent retrieval of previously-stored spatial information [60]. However, the small number of Grik4-Cre/DCC^*fl/fl*^ mice assessed using the Barnes Maze test limits the power of these data (Control, *n* = 10; Grik4-Cre/DCC^*fl/fl*^, *n* = 6). Further examination in future studies is required to fully elucidate the functional contribution of DCC in CA3 pyramidal neurons to spatial learning processes.

### The role of presynaptic DCC in synaptic transmission and plasticity

DCC plays an important role in SNARE-mediated vesicular exocytosis [30, 61, 62], however, this has not previously been demonstrated to impact synaptic transmission. The cytoplasmic tail of DCC directly binds TRIM9, which binds the synaptic vesicle t-SNARE, SNAP25 [30, 31, 63]. Additionally, netrin-1/DCC signaling can trigger vesicular fusion and exocytosis through the t-SNARE syntaxin-1, which mediates vesicular fusion and is essential for netrin-1-mediated axonal pathfinding in commissural neurons [64]. Interestingly, syntaxin-1 forms a complex with SNAP25 and synaptobrevin-2 that drives action potential-mediated vesicular fusion at synapses [65]. In the absence of presynaptic DCC, our findings indicate that a large proportion of spontaneous neurotransmission remains intact, whereas strong evoked neurotransmission is impaired, possibly due to altered action potential-mediated t-SNARE function, which may then result in reduced efficacy of vesicular exocytosis. Further, we did not observe a significant alteration in the mean amplitude of sEPSC events, consistent with work from other groups showing that spontaneous and evoked vesicular pools are regulated differently [66–68]. Fast vesicular exocytosis during evoked stimulation requires simultaneous activation of multiple SNARE complexes, whereas spontaneous exocytosis is thought to depend on a reduced stochiometric requirement of SNARE complex activation [69, 70]. We speculate that the lack of DCC may compromise t-SNARE activation, resulting in a reduction in the number of vesicles docked and released from the presynaptic terminal [30, 31]. Intriguingly, we detected no change in paired-pulse ratio in either R4ag11-Cre/DCC^*fl/fl*^ or Grik4-Cre/DCC^*fl/fl*^, which has been traditionally associated with assessment of changes in presynaptic release probability. Our findings suggest that release dynamics are not perturbed following presynaptic DCC deletion, however, the mechanism underlying the impact of DCC on neurotransmitter release remains unclear. Indeed, the lack of a change in sEPSC amplitude and frequency, or in paired-pulse ratio, following DCC deletion from CA3 neurons suggest the possibility that DCC may regulate increased vesicular mobilization during evoked activity without affecting single vesicular fusion events [68]. Our findings suggest that DCC may mediate large-scale presynaptic neurotransmitter vesicle mobilization to impact synapse function and plasticity [34]. Reduced evoked transmitter release may decrease the ability of Schaffer collateral synapses to initiate post-synaptic depolarization and activate NMDARs, resulting in the attenuation of LTP [71].

Postsynaptic modification of AMPARs plays a critical role in the expression of HFS-induced LTP, yet activity also induces changes in the volume and composition of the presynaptic compartment at Schaffer collateral synapses [72]. Indeed, presynaptic boutons exhibit enlargement comparable to that observed in postsynaptic spines following plasticity induction, and this enlargement coincides with an increase in the number of synaptic vesicles. Enlargement of presynaptic boutons can be induced by glutamate photo-uncaging, suggesting a possible retrograde postsynaptic signal to the presynaptic terminal [72]. We have previously demonstrated post-synaptic secretion of netrin-1 in response to NMDAR activation to facilitate HFS-induced LTP [7]. Together, these findings suggest that HFS may trigger post-synaptic secretion of netrin-1 that then directs co-ordinated DCC-dependent pre- and post-synaptic changes that underlie the expression of LTP.

### DCC and spatial memory consolidation

Repetitive training can promote dendritic spine formation and clustering in vivo [73, 74]. Consistent with a role governing dendritic spine dynamics mediated during learning, DCC signaling potently regulates F-actin cytoskeletal remodeling during development, and may serve a similar function at dendritic spines in the mature nervous system [6, 75]. DCC activates PLC-mediated L-type Ca^2+^ and canonical transient receptor (TRPC) channels to increase intracellular Ca^2+^, which critically regulates spine formation [28, 76–78]. Activation of both L-type Ca^2+^ channels and TRPCs have been implicated in synaptic plasticity underlying spatial memory, and genetic deletion of these channels can alter performance on hippocampal-dependent memory tasks [79–82]. Indeed, bath application of netrin-1 can depolarize developing growth cones through activation of both L-type Ca^2+^ channels and TRPCs [76, 77, 83]. Deletion of postsynaptic DCC may limit or occlude netrin-1 mediated activation of L-type Ca^2+^ channels and TRPCs, resulting in hyperpolarization of membrane potential as well as occlusion of Ca^2+^-mediated de novo spine formation or modification of existing dendritic spines. Additionally, deletion of DCC may limit PLC activation which, in addition to controlling membrane potential, may also influence plasticity associated with memory formation [84]. In contrast, deletion of DCC from CA3 neurons may limit Schaffer collateral synaptic plasticity underlying memory formation, resulting in spatial memory deficits. Consequently, conditional deletion of DCC from either CA1 or CA3 pyramidal neurons, which make essential contributions to spatial cognition, may impair structural plasticity and dendritic spine maturation associated with learning and memory formation required for spatial memory tasks.

## Supplementary information

**Additional file 1.**

## Data Availability

The datasets used and/or analysed during the current study are available from the corresponding author on reasonable request.
