# Supplementary material for: Pre- and post-synaptic roles for DCC in memory consolidation in the adult mouse hippocampus
Source: Mol Brain. 2020 Apr 7;13:56. doi: 10.1186/s13041-020-00597-2 (PMC7137442; doi:10.1186/s13041-020-00597-2)
Supplement: Supplementary file 1 — Additional file 1. [file 13041_2020_597_MOESM1_ESM.docx]

**Supplemental figure 1.**

**
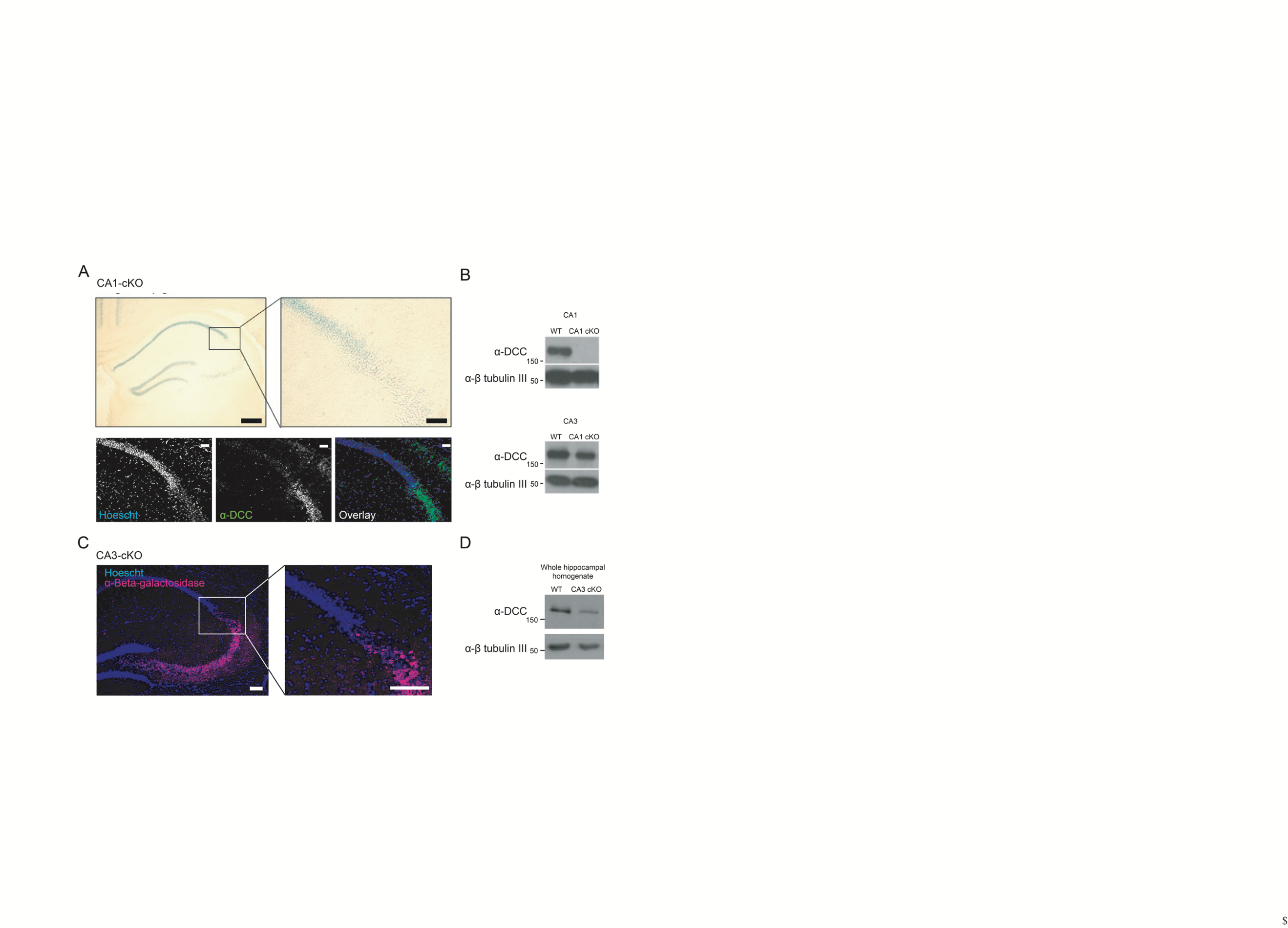
**

**Supplemental figure 1.** (**A**) Representative images of the hippocampus from a R4ag11-Cre-*ROSA26-lacZ* mouse showing X-gal staining. High magnification of area highlighted in box show X-gal stain (upper right), as well as a magnification of Hoescht stain (bottom left) and DCC immunoreactivity (bottom right) in brain section from adult R4ag11-Cre/DCC*^fl/fl^* mice. Scale bars are 200 µm for upper left, 100 µm for upper right, and 50 µm for bottom row. (**B**) Representative western blots from CA1 (top) and CA3 (bottom) showing DCC immunoreactivity in R4ag11-Cre/DCC*^fl/fl^* mice and age-matched control littermates. (C) Low- (left) and high- (right) magnification images of a coronal section of the CA1, CA2, and CA3 regions of hippocampus from a Grik4-Cre/DCC*^fl/fl^* mouse showing Hoechst stain (blue) and β-galactosidase immunoreactivity (magenta). Scale bar = 100 µm. (**D**) Representative western blot from whole hippocampal homogenates derived from Grik4-Cre/DCC*^fl/fl^* mice and their age-matched control littermates showing DCC immunoreactivity.
